# Survivorship in prostate cancer following robotic assisted radical prostatectomy–the time to act is now!

**DOI:** 10.1038/s41391-022-00589-4

**Published:** 2022-09-05

**Authors:** Findlay MacAskill, Majed Shabbir, Arun Sahai, Guy’s Post Pelvic Surgery (PPS) Research Group

**Affiliations:** 1 Department of Urology, Guy’s and St Thomas’ NHS Foundation Trust, London, UK; 2 King’s College London, London, UK

**Keywords:** Prostate cancer, Outcomes research

Most patients diagnosed with prostate cancer tend to have local disease and survive for more than 10 years following surgery. Preparations for cancer survivorship must begin at diagnosis and should not have a set endpoint. Despite advances in robotic-assisted radical prostatectomy (RARP), studies still report significant treatment regret exists, largely centred on the disparity between the expectation of better functional recovery and the actual functional outcome achieved [1]. In addition to the physical impact of surgery, the impact of functional changes on psychosocial wellbeing and relationships can be significant and is often overlooked. It is important that personalised care pathways ameliorate the patients’ cancer experience and provides them with a roadmap back to living life as normal.

Despite this, there are no specific RARP cancer survivorship guidelines in the UK and Europe, with management guidelines focusing primarily on oncological outcomes. The American Cancer Society (ACS) Prostate Cancer Survivorship guidelines were published in 2014 and the NHS launched the National Cancer Survivorship Initiative: A ‘how to guide’ (NCSI) in 2015 with generic advice on how to approach building pathways [2, 3]. Recommendations were primarily based on expert opinion, but recent work by Dunn et al utilised patient input to structure RARP care pathways [4]. Little is known about the structure of UK or European Centres’ post RARP care pathways and this uncertainty leads to variations in care, patient’s survivorship outcomes and treatment experience.

There are several core areas that need to be considered and addressed when designing and delivering post RARP care promoted by ACS, NCSI and Dunn et al [2–4]. These include;

## Patient-centred care

A RARP pathway should be structured to be personalised to the need of the patient. Prior to any decision to undergo RARP, a detailed functional assessment should occur and personalised risk factors for poorer outcomes explained. Current research should focus on creating better pre-operative predictive nomograms to generate improved decision tools that can aid understanding of potential outcomes. Furthermore, we should utilise data already obtained, such as the pre-biopsy MRI, to investigate if information gained relates to outcomes [5].

A useful tool we have developed to aid empowerment is a ‘care passport’, similar to an antenatal record. This passport becomes a core document containing records of functional, psychological and oncological outcomes during the cancer treatment pathway, initiated before surgery. With a better educated, more resilient, and informed patient, the burden on the healthcare provider should be reduced as patients have many of the tools required to aid their recovery. They can be better supported even with more limited healthcare provider input [4].

## Health promotion

Healthcare information needs to tailor to the individual. The survivorship programme should adapt as the patients’ survivorship experience evolves. The program should focus on specific areas of functional recovery relevant to their surgery but should also focus on their general physical and psychological wellbeing, to provide opportunistic holistic care. Improving personal health and performance status improves quality of life through reducing fatigue, anxiety and depressive symptoms [2]. Information provided needs to accommodate health literacy and patients’ preferred route to access healthcare. Work should focus on improving access to those from LGBTQ + communities and those from minority cultures to ensure equity in healthcare outcomes.

## Surveillance

Following RARP, the rehabilitation phase of up to two years should not mark the end of aftercare. Rather than just having remote PSA monitoring, patients should have yearly survivorship ‘check-ins’, focussing on; [2, 4].

- maintenance of mental health
- monitoring and managing the physical effects of treatment
- surveillance of any co-morbidities
- assessing for any disease recurrence

The use of validated patient reported-outcomes measures (PROMs) can assist in healthcare surveillance, but should be used in a way that impacts patient care, rather than just informing on research [2, 6]. There has been a positive increase in use of PROMs to track survivorship care but the ones in current use do not fully encompass the entirety of the survivorship experience [7]. Additionally, much needed work is still required to ensure PROMs is relevant to minorities as well. Only by producing holistic PROMs will we truly capture the impact of treatment of functional recovery.

## Communication

The benefits of increasing digitisation of healthcare are clear, careful attention should be given to those without access to the internet or smartphones. Digital literacy should not be a new barrier for quality of life. Access to the service should be through multiple avenues of communication and there should be timely expert response to queries.

## National outcomes measurements

There is a shift to monitoring more than oncological outcomes and for better reporting of continence and sexual dysfunction following RARP. It is also vital that this data is registered in national databases. Outcome data should be freely available and there should be structures in place for teams to learn from better performing centres. The goal should be to have clearly defined standards nationally, with an infrastructure in place to ensure the provision of an equitable and high standard of care irrespective of geography.

## Evidence-based Survivorship Interventions

It is essential that patients are supported with the best evidence-based interventions to ensure maximal chance of attaining their quality-of-life. An example of areas to be improved was showcased in the EUROPROMs study [8]. It showed that a small percentage of patients had tried the different erectile dysfunction management options following prostate cancer treatment. It is essential that management of the expectant side effects of surgery, are standardised with quick escalation of treatments. This will give patients the best chance irrespective of where, or by whom, they are treated.

## Conclusion

It has been eight years since ACS published its initial survivorship guidelines, and 7 years since the NHS NCSI launched its initiative. During this time, the diagnosis and management of prostate cancer has advanced greatly on the wings of well-funded research and technological innovation, but we are still way behind on defined guidance on optimised post RARP care. The development of clear standardised guideline recommendations for RARP survivorship care are essential to improve quality of life outcomes and ensure equitable, high quality clinical care.

### Supplementary information

Members of Guy's Post Pelvic Surgery Research Group
