# Supplementary material for: Survivorship in prostate cancer following robotic assisted radical prostatectomy–the time to act is now!
Source: Prostate Cancer Prostatic Dis. 2022 Sep 5;27(1):46–7. doi: 10.1038/s41391-022-00589-4 (PMC10876470; doi:10.1038/s41391-022-00589-4)
Supplement: Supplementary file 1 — Members of Guy's Post Pelvic Surgery Research Group [file 41391_2022_589_MOESM1_ESM.docx]

Members of the GSTT PPS Study Group:

Findlay MacAskill 1,2,

Arun Sahai 1,2,

Majed Shabbir 1,2,

Prokar Dasgupta 1,2

Tet Yap 1,2

Paul Cathcart 1

Amy Sandher 1

Karen Briggs 1

Christian Brown 1

Jonathan Noel 1

Ben Challacombe 1,2

Rick Popert 1

Raveen Sandher 1

Claire Taylor 1

Sachin Malde 1

^1^ Department of Urology, Guy’s and St Thomas’ NHS Foundation Trust, London, UK

^2^ King’s College London, UK
